# Educational inequality in Rio de Janeiro and its impact on multimorbidity: evidence from the Pró-Saúde study. A cross-sectional analysis

**DOI:** 10.1590/1516-3180.2017.0209100917

**Published:** 2018-03-05

**Authors:** Adelson Guaraci Jantsch, Ronaldo Fernandes Santos Alves, Eduardo Faerstein

**Affiliations:** I MD, MSc. Doctoral Student, Instituto de Medicina Social, Universidade do Estado do Rio de Janeiro (UERJ), and Coordinator, Residency Program in Family and Community Medicine, Secretaria Municipal de Saúde do Rio de Janeiro, Rio de Janeiro (RJ), Brazil.; II MSc. Doctoral Student, Instituto de Medicina Social, Universidade do Estado do Rio de Janeiro (UERJ), Rio de Janeiro (RJ), Brazil.; III MSc, PhD. Associate Professor, Instituto de Medicina Social, Universidade do Estado do Rio de Janeiro, Rio de Janeiro (RJ), Brazil.

**Keywords:** Comorbidity, Health status disparities, Educational status, Cross-sectional studies

## Abstract

**BACKGROUND:**

Information about multimorbidity is scarce in developing countries. This study aimed to estimate the association of educational attainment with occurrences of multimorbidity in a population of public employees on university campuses in Rio de Janeiro.

**DESIGN AND SETTING:**

We conducted cross-sectional analyses on baseline data (1999-2001) from 3,253 participants in the Pró-Saúde study, conducted in Brazil.

**METHODS:**

The prevalence of multimorbidity, defined as a self-reported history of medical diagnoses of two or more chronic conditions, was estimated according to sex, age, smoking, obesity and educational level. The association between education and multimorbidity was estimated using odds ratios (OR) and the relative and slope indices of inequality, in order to quantify the degree of educational inequality among individuals with multimorbidity in this population.

**RESULTS:**

Greater age, female sex, smoking and obesity had direct associations with multimorbidity; and tobacco exposure and obesity also showed direct relationships with poorer educational level. There was a monotonic inverse linear trend between educational level and the presence of multimorbidity among women, with twice the odds (OR 2.47; 95% confidence interval, CI: 1.42-4.40) between extremities of schooling categories. There was excess multimorbidity of 22% at the lowest extremity of schooling, thus showing that women with worse educational status were more affected by the outcome. No trend and no excess multimorbidity was seen among men.

**CONCLUSIONS:**

Educational inequality is an important determinant for development of multimorbidity. Men and women experience its effect differently. Researchers need to consider that sex may be an effect modifier in multimorbidity studies.

## INTRODUCTION

Multimorbidity, defined as the coexistence of two or more chronic conditions in the same patient,^1^^,^^2^^,^^3^ has been consistently correlated with several outcomes, such as greater use of healthcare services,^3^ poor quality of life,^4^ polypharmacy and adverse drug events,^5^ functional decline,^6^ disability,^7^ hospitalization^8^ and higher risk of death.^9^ This concept changes the way of looking at co-occurrence of multiple diseases in a single person. Traditionally, the approach taken in cases of patients with multiple conditions has been that they have one protagonist disease that is influenced by a group of concurrent conditions (comorbidities). In multimorbidity, there are no main diseases. All of them have their own importance within the patient’s clinical picture.

Most studies on multimorbidity have been conducted in high-income countries (HIC) in Europe, North America and Australia, where it affects the majority of the population older than 65 years.^10^ Like in many single diseases and risk factors, age is the main determinant for its occurrence. Moreover, studies have shown that multimorbidity is a major topic of concern relating to health inequalities^11^^,^^12^ because of its association with low socioeconomic position, measured via household income^13^ or educational attainment.^14^ Proximal determinants, such as obesity,^15^ smoking, alcohol consumption and lack of physical activity have also been identified.^16^

Evidence is scarce regarding its determinants and impact in low and middle-income countries (LMIC), where population dynamics, social development, epidemiological patterns and healthcare services may differ from those in HIC.^17^^,^^18^ Some studies in LMIC have also shown an association between low socioeconomic position and multimorbidity,^19^^,^^20^^,^^21^ but the determinants of multimorbidity are still insufficiently studied in LMIC.^22^^,^^23^

The few studies conducted in Brazil were restricted to specific populations. They showed that multimorbidity was associated with higher risk of death and readmission after hospital discharge, among elderly people.^24^ It was also associated with aging and obesity and with worse self-perception of health among women;^25^^,^^26^ and it was highly prevalent among elderly people using public primary healthcare services.^27^

In addition, several methodological issues relating to studies on multimorbidity are still unresolved.^1^^,^^28^ These include what the best way to measure it would be,^29^ which clusters of diseases are more harmful^30^ and what role sex has in relation to development of multimorbidity.^31^

The aims of the present study were to determine the prevalence of multimorbidity in a population of adult Brazilians and to investigate the relationship between its occurrence and socioeconomic position, measured via educational attainment.

## METHODS

### Study design

Our study was a cross sectional analysis that was carried out using data from the baseline of the Pró-Saúde study (PSS). PSS is a longitudinal investigation program on public employees on university campuses in Rio de Janeiro, Brazil, who were not members of the teaching staff. It has mainly focused on social determinants of health.^32^

This study was approved by the Research Ethics Committee of Pedro Ernesto University Hospital (record 224/1999 and record 461/2001).

### Data collection

Between 1999 and 2012, four phases of data collection were conducted, including self-completion of questionnaires, anthropometric measurements and other tests. The PSS baseline was composed of 3,253 subjects who formed part of the inventory both in phase 1 (1999) and in phase 2 (2001-2). 

Pregnant women and people over 69 years of age were considered ineligible for this study. These exclusions left the sample with 3,251 individuals.

PSS data were collected using a multidimensional, self-completed questionnaire administered in the workplace. A pilot study and test-retest reliability studies, and independent double data entry, were performed to ensure data quality.^33^

### Outcome

Self-reported information about chronic medical conditions was obtained both from the 1999 and from the 2001 data collection phases through the following question: “Have you ever been informed by any physician that you had...?”. This question was applied to a list of 14 different clinical conditions: hypertension, diabetes, dyslipidemia, angina, ischemic heart disease, stroke, asthma, chronic obstructive pulmonary disease, cholecystitis, peptic ulcer, repetitive strain injury, osteoarthritis, hyperthyroidism and hypothyroidism. Each condition only had two response options (yes/no). Hypertension, diabetes, dyslipidemia, stroke, cholecystitis, peptic ulcer, repetitive strain injury and osteoarthritis were considered to be single diseases. Angina and ischemic heart disease were aggregated into one condition, labeled as “coronary heart disease”. Asthma and chronic obstructive pulmonary disease were aggregated as “lung condition”. Hyperthyroidism and hypothyroidism were aggregated as “thyroid condition”. The dependent variable of multimorbidity was defined as a self-reported medical diagnosis of two or more of these 11 chronic conditions in the same person.^2^

### Study variables

Our main exposure of interest was education attainment, as a marker of individual socioeconomic status. Data were collected in 1999 in seven different categorical levels: incomplete elementary school, completed elementary school, incomplete high school, completed high school, incomplete university, completed university and postgraduate studies. Sex, smoking status, obesity and age were studied as intervenients of the process, and these data were collected in 2001. The respondent was considered to have been exposed to tobacco if he/she answered “yes, I am exposed to tobacco” or “I used to be, in the past, but not anymore”. The option “never have been” was considered to represent non-exposure. Obesity was evaluated by means of a double measurement of abdominal circumference at the level of the navel and was defined according to abdominal circumference thresholds of 88 cm for women and 102 cm for men, as suggested by the World Health Organization (WHO).^34^ Age was used as a discrete variable, in years.

### Statistical analysis

Frequencies of multimorbidity, smoking status and obesity were calculated according to sex and for each educational level. Prevalences and 95% confidence intervals were calculated using the chi-square test for linear trend, regarding educational levels and multimorbidity. We performed these analyses for the whole population and stratified according to sex.

Age-adjusted logistic regression models were built to examine predictors of multimorbidity (model 1). We then explored the mediating effects of smoking status and obesity in the age-adjusted models (model 2). Adjusted odds ratios and 95% confidence intervals for multimorbidity on each educational level were calculated for both models.

Finally, we estimated the degree of educational inequality in occurrences of multimorbidity by calculating the slope index of inequality (SII) and relative index of inequality (RII). These indices have the capacity to produce absolute and relative estimates of the socioeconomic gradient relating to health, and they are based on weighted linear and logistic regression analysis, respectively.^35^^,^^36^

For this calculation, the exposure was transformed from an ordinal categorical variable into a continuous variable, composed of a series of numerical values between 0 and 1 that represented the median of the cumulative interval in each category. Each new value was used in the related regression models according to the numerical score, which represented the proportional size of the population in each category, taking into account the information from all simultaneous levels and the relative positions of the value within the population scale.

In this manner, SII and RII provide measurements of the relationship between socioeconomic status and the health outcome that are more reliable. Since 2013, the World Health Organization has recommended that SII and RII should be used as indicators for reporting on health inequalities. These indices make the notions of excess and relative risk between two hypothetical extremities more reliable and comprehensible than do the traditional measurements (odds ratios, OR, and relative risk, RR), through log-linear and linear models, respectively.^36^^,^^37^

All analysis were performed using the R statistical package (version 3.3.1) and P-values < 0.05 were defined as statistically significant.

## RESULTS

All of the 3,251 subjects at the baseline answered the questions about their educational level and filled out the morbidity inventory. 107 subjects did not provide information about their tobacco status and 52 did not have their abdominal circumferences measured, thus totaling 159 subjects with missing data. There were no differences between these 159 subjects and the other 3,092 regarding educational attainment and the prevalence of multimorbidity. Therefore, we considered this to be a situation of random missing data. Hence, the analytical sample comprised 3,092 subjects aged between 24 and 69 years. There were 1,378 men (44.6%) and 1,714 women (55.4%), with average ages of 41.3 years (95% confidence interval, CI 40.9-41.8) and 42.5 years (95% CI: 42.2-42.9), respectively.

The women had higher educational levels than the men and for both sexes, people with high levels of education tended to be younger. The women had a lower rate of tobacco use, such that 39.8% of them had used it during their lifetime, versus 45.1% of the men. However, obesity was more common among the women (43.6%) than among the men (24.2%). The prevalence of multimorbidity was 27.3% among the men and 37.8% among the women. These data are summarized in Table 1.

**Table 1:** Descriptive analysis on the Pró-Saúde study baseline (1999-2001), Rio de Janeiro

	General		Men		Women	
	N 3,092	% 100	N 1,378	% 44.6	N 1,714	% 55.4
Age						
Mean	42.00 (41.70-42.30)		41.33 (40.9-41.8)		42.55 (42.2-42.9)	
Minimum-maximum	24-69		24-68		25-69	
Education						
Postgraduate	515	16.7	170	12.3	345	20.1
Complete university	803	26.0	325	23.6	478	27.9
Incomplete university	433	14.0	204	14.8	229	13.4
Complete high school	659	21.3	306	22.2	353	20.6
Incomplete high school	274	8.9	145	10.5	129	7.5
Complete elementary	178	5.8	96	7.0	82	4.8
Incomplete elementary	230	7.4	132	9.6	98	5.7
Tobacco exposure						
Smoker	675	21.8	335	24.3			340	19.8
Former smoker	629	20.3	286	20.8			343	20.0
Never smoker	1,788	57.8	757	54.9			1,031	60.2
Obesity								
Obese	1,082	35.0	334	24.2					748	43.6
Non-obese	2,010	65.0	1,044	75.8					966	56.4

The associations between educational level and presence of chronic conditions according to sex are shown in Table 2. Hypertension, dyslipidemia, repetitive strain injury and osteomuscular problems were the most common chronic conditions reported by both men and women. Every condition analyzed in this study, except repetitive strain injury, showed a significant linear trend among the women, such that these conditions were always more prevalent among those with low educational levels. The men showed a less pronounced trend among educational levels, which was statistically significant only for hypertension, diabetes, coronary heart disease, repetitive strain injury and peptic ulcer. Stroke and thyroid disease did not present any trend among the men and very low prevalence as well. Subjects in the highest education categories were less exposed to tobacco over the courses of their lives, with the same linear trend for both sexes. The same educational gradient was observed in relation to obesity among the women, but not among the men.

**Table 2:** Prevalence of morbidities, tobacco exposure, obesity and multimorbidity according to educational level at the Pró-Saúde study (PSS) baseline (1999-2001), Rio de Janeiro (RJ), Brazil

	Total	Educational level							P-value
		Postgraduate studies	Complete university	Incomplete University	Complete high school	Incomplete high school	Complete elementary	Incomplete elementary	
Men									
Hypertension	28.2	27.6	17.2	28.4	30.7	37.9	31.2	36.4	< 0.001
Diabetes	5.5	4.7	1.5	2.9	6.5	9.7	9.4	10.6	< 0.001
Dyslipidemia	22.0	25.9	18.1	19.6	21.9	28.3	21.9	23.5	0.37
Coronary heart disease	2.4	1.8	0.9	1.0	2.0	2.1	8.3	6.1	< 0.001
Stroke	0.6	1.2	0.6	0.0	0.3	0.0	3.1	0.0	0.95
Lung condition	2.1	2.3	2.5	2.4	2.0	2.1	2.1	0.8	0.31
Peptic ulcer	5.3	2.3	3.1	4.4	4.9	9.7	11.5	7.6	< 0.001
Cholecystitis	2.2	3.5	1.5	2.0	1.3	2.8	4.2	2.3	0.78
Repetitive strain injury	10.3	12.3	12.6	11.3	9.5	10.3	4.2	6.8	< 0.001
Osteomuscular	7.8	8.2	5.2	4.4	9.1	11.0	8.3	12.1	0.01
Thyroid disease	0.4	0.0	0.6	0.5	0.3	0.7	1.0	0.0	0.86
Tobacco exposure	45.1	37.6	30.5	34.8	48.0	60.0	70.8	64.4	< 0.001
Obesity	24.2	27.1	18.5	27.9	26.1	24.8	22.9	25.0	0.55
Multimorbidity	23.7	22.9	17.5	22.1	23.8	33.1	27.1	28.8	0.002
Women									
Hypertension	30.6	14.8	18.4	30.1	36.0	48.1	67.1	74.5	< 0.001
Diabetes	4.9	1.7	2.5	3.5	5.1	10.1	15.8	14.3	< 0.001
Dyslipidemia	24.0	19.1	21.1	24.9	21.8	31.0	30.5	46.9	< 0.001
Coronary heart disease	2.6	1.4	1.3	0.4	2.5	70.	6.1	10.2	< 0.001
Stroke	0.7	0.3	0.2	0.4	0.8	2.3	2.4	1.0	0.009
Lung condition	4.7	2.9	3.6	5.7	4.2	10.8	4.9	8.2	0.002
Peptic ulcer	5.5	3.2	4.6	3.9	6.5	9.3	12.2	8.2	< 0.001
Cholecystitis	6.2	5.2	4.6	5.2	6.5	5.4	15.8	12.2	< 0.001
Repetitive strain injury	23.6	22.3	19.2	32.3	25.2	23.3	20.7	25.5	0.25
Osteomuscular	1.6.0	7.2	8.6	14.8	19.3	22.5	39.0	46.9	< 0.001
Thyroid disease	4.9	6.1	6.3	3.1	3.1	7.0	3.7	3.1	0.08
Tobacco exposure	39.8	33.9	35.6	38.9	41.6	59.7	45.1	46.9	< 0.001
Obesity	43.6	32.8	34.1	41.9	51.0	54.3	67.1	72.4	< 0.001
Multimorbidity	34.2	22.6	21.7	33.6	37.7	51.9	68.3	72.4	< 0.001
Whole population									
Multimorbidity	29.5	22.7	20.0	28.2	31.2	42.0	46.1	47.4	< 0.001*

*P-value for linear trend.

The association between educational level and multimorbidity differed between the men and women and can be seen in Table 3. The women showed a linear gradient across the educational levels, such that those with incomplete elementary school presented more than twice the chance of occurrences of multimorbidity in relation to those who had done postgraduate studies (OR = 2.77; 95% CI: 1.61-4.91). On the other hand, the men did not show any gradient in the age-adjusted model, such that there was a lower chance of multimorbidity among those with incomplete and complete elementary school, but this was not statistically significant.

**Table 3:** Age and multivariate adjusted odds ratio (OR, 95% confidence interval [CI]) and slope index of inequality and relative index of inequality for multimorbidity according to educational level among men and women at the Pró-Saúde study baseline (1999-2001), Rio de Janeiro (RJ), Brazil

	Women		Men	
	Age-adjusted	Multivariate adjusted OR (95% CI)	Age-adjusted	Multivariate adjusted OR (95% CI)
Obesity	1.90* (1.51-2.39)		2.19* (1.65-2.90)
Tobacco	1.27* (1.01-1.59)		1.20 (0.91-1.59)
Education				
Postgraduate studies	1	1	1	1
Complete university	0.85 (0.60-1.0)	0.83 (0.59-1.19)	0.84 (0.53-1.37)	0.90 (0.56-1.47)
Incomplete university	1.92^†^ (1.29-2.85)	1.79* (1.21-2.68)	1.15 (0.69-1.91)	1.11 (0.66-1.86)
Complete high school	1.43^‡^ (1.01-2.05)	1.33 (0.92-1.90)	0.90 (0.57-1.44)	0.91 (0.57-1.46)
Incomplete high school	2.05^†^ (1.30-3.24)	1.85^†^ (1.16-2.94)	1.24 (0.74-2.09)	1.26 (0.74-2.15)
Complete elementary	3.6* (1.74-5.46)	2.75* (1.56-4.92)	0.66 (0.36-1.21)	0.69 (0.37-1.28)
Incomplete elementary	2.77* (1.61-4.91)	2.47* (1.42-4.40)	0.68 (0.39-1.19)	0.70 (0.40-1.24)
Slope index of inequality		22.03* (14.2-29.8)		-3.4 (-11.6-4.8)
Relative index of inequality		2.97* (1.94-4.54)		0.74 (0.45-1.23)

P-value: * ≤ 0.001; ^†^≤ 0.01; ^‡^≤ 0.05.

Since obesity and smoking were influenced by socioeconomic position and had an impact on the occurrence of the outcome, they were considered, along with age, to be potentially intervening factors in the relationship between education and multimorbidity. However, their presence in the age-adjusted models only slightly decreased the association between low educational level and occurrences of multimorbidity among the women. Among the men, the same pattern of no tendency across the social gradient was observed. Although the women had higher prevalence of obesity and a marked gradient across all educational levels, the point estimate of its effect on occurrences of multimorbidity was greater among the men (for men, OR = 2.19; 95% CI: 1.65-2.90; versus for women, OR = 1.90; 95% CI: 1.51-2.39). The opposite association was seen in relation to tobacco exposure, such that the men presented higher prevalence, but the association with multimorbidity was statistically significant only among the women (for men, OR = 1.27; 95% CI: 1.01-1.59; versus for women, OR = 1.20; 95% CI: 0.91-1.59).

SII and RII were then calculated using the same models. For the women, the excess multimorbidity at the lowest educational level was 22%, in comparison with the highest level (SII = 22.03; 95% CI: 14.2-29.8). In relative terms, there was a three times greater chance of presenting multimorbidity at the lowest extremity in relation to the highest educational level (RII = 2.97; 95% CI: 1.94-4.54), thus showing that there was a high level of inequality among the women in this population. For the men, after adjustment for age, smoking status and obesity, there was no statistical difference in either index across the population (RII = 0.74; 95% CI: 0.45-1.23; and SII = -3.4; 95% CI: -11.6-4.8).

## DISCUSSION

To the best of our knowledge, this was the first study to describe the impact of educational inequality on multimorbidity in an adult population in Brazil. Despite difficulties that have been mentioned in systematic reviews regarding comparisons of the prevalences of multimorbidity in different countries,^7^^,^^38^ the prevalence of multimorbidity of 33.1% that we observed in our study was close to the prevalences found in developed countries such as the Netherlands,^39^ Canada^13^ and Australia,^40^ but it was considerably higher than in other LMIC countries.^19^^,^^20^^,^^21^

As expected, the prevalence of multimorbidity increased with age and was higher among women than among men, across educational levels. Both obesity and smoking status showed high prevalence in the study population and these had significant relationships with the outcome, albeit in different ways. The men tended to be more exposed to tobacco over the course of their lives, but the effect of tobacco exposure on multimorbidity was perceived especially among the women. On the other hand, the women tended to be more obese, with a clear social gradient, but the effect of obesity on multimorbidity was stronger among the men. This kind of information is very important for understanding the paths that are followed until occurrences of multimorbidity are observed.

Hypertension, dyslipidemia, repetitive strain injury and osteomuscular problems were the most prevalent chronic conditions reported both by men and women. However, regarding the distribution of these problems across the different socioeconomic statuses, the women showed marked trends towards high prevalence of almost every chronic condition, among those at the lowest educational level, except in relation to repetitive strain injury and thyroid diseases. Among the men, this trend was only noticeable in relation to hypertension, diabetes, coronary heart disease and peptic ulcer, but not with the same intensity as seen among the women. Stroke showed very low prevalence in this study, thus making it impossible to infer any tendency across the population.

The association between educational level and multimorbidity differed substantially regarding sex in the multivariate adjusted models. Among the women, there was a monotonic and inverse linear trend between educational level and occurrences of multimorbidity, with a threefold greater chance (RII = 2.97; 95% CI: 1.94-4.54) of the outcome between the extremities of schooling. The SII revealed that there was excess prevalence of multimorbidity of 22% among the women at the lowest educational level.

Education is a lifelong process and a lack of opportunities for receiving education represents a considerable disadvantage for children’s health.^41^ People who are less educated and/or spent their childhoods living in poor socioeconomic conditions have worse health outcomes than do people who had access to education, enjoyed good housing conditions and did not experienced food insecurity during the first years of their lives. The major concern of the WHO Commission on Social Determinants of Health^42^ is that developing countries have a considerable educational gap between rich and poor people, and an even larger gap between men and women.

Our results agree with those observed in several other studies, in which educational inequality played an important role in the development of multimorbidity.^20^^,^^43^^,^^44^^,^^45^ But the remarkable fact in the present study is that this trend was detected only among women. Among men, no trend across the social gradient was observed (RII 0.74; 95% CI: 0.45-1.23; and SII = -3.4; 95% CI: -11.6-4.8).

It was beyond the scope of the present study to explore the singularities of the prevalence, determinants and burden of multimorbidity relating to differences between the sexes. Nonetheless, the findings showed different effects on the outcome from exposures and interve­nients, which raises some important questions about this subject, especially relating to the way in which measurements are made.

We could easily infer from our data that educational inequality is unrelated to occurrences of multimorbidity in the male population. However, recent evidence in the literature^14^^,^^20^^,^^44^ shows that this statement lacks plausibility. This makes us look at this issue from another angle, which is: why did the women in this study show a monotonic and inverse linear trend between educational level and the presence of multimorbidity, but the men did not?

The prevalence of multimorbidity is absolutely intertwined with the way in which it is measured. Differences within^38^ and between similar countries^28^ can sometimes be of considerable magnitude in different studies. One possible explanation for this sex-related pattern concerns the historical time at which the data were collected. Between 1999 and 2001, i.e. the period during which the PSS baseline data were collected, the city of Rio de Janeiro already had a very extensive hospital network, albeit disorganized and disjointed with the fledgling system of primary healthcare. At that time, the Family Health Strategy (Brazilian federal policy for primary healthcare) coverage in the municipality was only 3%, which was the lowest among Brazilian state capitals.^46^ If access is the first attribute of primary healthcare,^47^ knowing the way in which these individuals accessed and used the reference healthcare services (if they had any) may be crucial for interpreting our findings.

Contact with a healthcare service is essential for a diagnosis to be made. The way in which morbidities are commonly measured, i.e. by asking “Have you ever been informed by any physician that you had...?”, makes it impossible for a person with no access to health services to answer this question. This would not be a matter of such concern if equity between the sexes regarding access to healthcare services existed, but this was not the case in Brazil in 2001^48^ and it is not the case in many LMIC,^42^^,^^49^ where specific health interventions like cervical cancer screening, prenatal care, contraception and child care may provide selective access for women, but not for men. Thus, women are more exposed to healthcare providers and they are therefore more likely to be diagnosed with a chronic condition. For example, the prevalence of multimorbidity was found to be only 4% among South African adults and 70% of them were women.^19^

For this reason, considering that the construct of multimorbidity measures not only the current health status of a population but also the health inequalities inherent to the healthcare system, it is crucial to understand the complexity of this phenomenon. Moreover, beyond these absolute inequalities, there is a relative difference among people relating to the way in which they interact with healthcare services, and this relates both to healthcare professionals and to patients. From this angle, the concept of “multimorbidity”, which is thought to be a good way to capture the current status of chronic conditions in a population, ends up also capturing the relationship between individuals and the healthcare services.

In addition to the inequalities in healthcare access that lead to differences in the prevalences of chronic conditions and multimorbidity between men and women, it may be also possible that these outcomes could have a closer relationship with women than with men. There is some evidence suggesting that people tend to develop different morbidity patterns, depending on their sex.^31^ Women might be more prone to develop chronic conditions that lead them to frailty and functional disability, while men could tend to develop chronic conditions that shorten their lives, i.e. leading them to premature death. If this is the case, different diseases would form the conditions of multimorbidity observed for each sex, but there would not be any differences in prevalence. This subject was not the focus of our study but, even if this phenomenon were to occur in this population, it would not explain why we did not see the same gradient of multimorbidity among men and women across educational levels.

Gender needs to be studied at least as an effect modifier, because of its different relationship with the outcome and the underlying way in which it is measured. The same reasoning applies to describing the healthcare system within which studies on multimorbidity are conducted. If there is a difference in the accessibility of healthcare services between men and women in a population, thereby representing an absolute inequality in health, the data analysis needs to be stratified according to sex. With unequal access to healthcare services, inferences about multimorbidity cannot be made in the same way that is done in studies conducted in high-income and countries and in those with strong primary healthcare. Nonetheless, we can use such inferences to take a better look at healthcare services and socioeconomic inequalities.

## CONCLUSION

The evidence gathered here is consistent with what is available in the literature, and it shows that educational inequality is an important determinant for the development of multimorbidity. However, contrary to findings from studies conducted in wealthier countries, men and women experienced this association differently, thus highlighting that gender needs to be taken into consideration as a potential effect modifier in future studies on multimorbidity.

## References

[1] Valderas JM, Mercer SW, Fortin M (2011). Research on patients with multiple health conditions: different constructs, different views, one voice. Journal of Comorbidity.

[2] Valderas JM, Sibbald B, Salisbury C, Salisbury C, Roland M (2009). Defining comorbidity: implications for understanding health and health services. Ann Fam Med.

[3] van Oostrom SH, Picavet HS, de Bruin SR (2014). Multimorbidity of chronic diseases and health care utilization in general practice. BMC Fam Pract.

[4] Fortin M, Lapointe L, Hudon C (2004). Multimorbidity and quality of life in primary care: a systematic review. Health Qual Life Outcomes.

[5] Calderón-Larrañaga A, Poblador-Plou B, González-Rubio F (2012). Multimorbidity, polypharmacy, referrals, and adverse drug events: are we doing things well?. Br J Gen Pract.

[6] Marengoni A, von Strauss E, Rizzuto D, Winblad B, Fratiglioni L (2009). The impact of chronic multimorbidity and disability on functional decline and survival in elderly persons. A community-based, longitudinal study. J Intern Med.

[7] Salive ME (2013). Multimorbidity in older adults. Epidemiol Rev.

[8] France EF, Wyke S, Gunn JM (2012). Multimorbidity in primary care: a systematic review of prospective cohort studies. Br J Gen Pract.

[9] Nunes BP, Flores TR, Mielke GI, Thumé E, Facchini LA (2016). Multimorbidity and mortality in older adults: A systematic review and meta-analysis. Arch Gerontol Geriatr.

[10] Tinetti ME, Fried TR, Boyd CM (2012). Designing health care for the most common chronic condition--multimorbidity. JAMA.

[11] Barnett K, Mercer SW, Norbury M (2012). Epidemiology of multimorbidity and implications for health care, research, and medical education: a cross-sectional study. Lancet.

[12] Orueta JF, Nuño-Solinís R, García-Alvarez A, Alonso-Morán E (2013). Prevalence of multimorbidity according to the deprivation level among the elderly in the Basque Country. BMC Public Health.

[13] Roberts KC, Rao DP, Bennett TL, Loukine L, Jayaraman GC (2015). Prevalence and patterns of chronic disease multimorbidity and associated determinants in Canada.

[14] Nagel G, Peter R, Braig S (2008). The impact of education on risk factors and the occurrence of multimorbidity in the EPIC-Heidelberg cohort. BMC Public Health.

[15] Booth HP, Prevost AT, Gulliford MC (2014). Impact of body mass index on prevalence of multimorbidity in primary care: cohort study. Fam Pract.

[16] Fortin M, Haggerty J, Almirall J (2014). Lifestyle factors and multimorbidity: a cross sectional study. BMC Public Health.

[17] Frenk J, Frejka T, Bobadilla J (1991). La transición epidemiológica en América Latina. Bol Of Sanit Panam.

[18] Duarte EC, Barreto SM (2012). Transição demográfica e epidemiológica: a Epidemiologia e Serviços de Saúde revisita e atualiza o tema. Epidemiol Serv Saúde.

[19] Alaba O, Chola L (2013). The social determinants of multimorbidity in South Africa. Int J Equity Health.

[20] Chung RY, Mercer S, Lai FT (2015). Socioeconomic Determinants of Multimorbidity: A Population-Based Household Survey of Hong Kong Chinese. PLoS One.

[21] Ha NT, Le NH, Khanal V, Moorin R (2015). Multimorbidity and its social determinants among older people in southern provinces, Vietnam. Int J Equity Health.

[22] Omran AR (2005). The epidemiologic transition: a theory of the epidemiology of population change. 1971. Milbank Q.

[23] Afshar S, Roderick PJ, Kowal P, Dimitrov BD, Hill AG (2015). Multimorbidity and the inequalities of global ageing: a cross-sectional study of 28 countries using the World Health Surveys. BMC Public Health.

[24] Sousa-Muñoz RL, Ronconi DE, Dantas GC, Lucena DMS, Silva IBA (2013). Impacto de multimorbidade sobre mortalidade em idosos: estudo de coorte pós-hospitalização [Impact of multimorbidity on mortality in elderly: a post-hospitalization cohort study]. Rev Bras Geriatr Gerontol.

[25] de S Santos Machado V, Valadares AL, Costa-Paiva LH (2013). Aging, obesity, and multimorbidity in women 50 years or older: a population-based study. Menopause.

[26] de Souza Santos Machado V, Valadares AL, da Costa-Paiva LS, Moraes SS, Pinto-Neto AM (2012). Multimorbidity and associated factors in Brazilian women aged 40 to 65 years: a population-based study. Menopause.

[27] Nunes BP, Thumé E, Facchini LA (2015). Multimorbidity in older adults: magnitude and challenges for the Brazilian health system. BMC Public Health.

[28] Diederichs C, Berger K, Bartels DB (2011). The measurement of multiple chronic diseases--a systematic review on existing multimorbidity indices. J Gerontol A Biol Sci Med Sci.

[29] Diederichs CP, Wellmann J, Bartels DB (2012). How to weight chronic diseases in multimorbidity indices? Development of a new method on the basis of individual data from five population-based studies. J Clin Epidemiol.

[30] van den Bussche H, Koller D, Kolonko T (2011). Which chronic diseases and disease combinations are specific to multimorbidity in the elderly? Results of a claims data based cross-sectional study in Germany. BMC Public Health.

[31] Verbrugge LM (1985). Gender and health: an update on hypotheses and evidence. J Health Soc Behav.

[32] Faerstein E, Chor D, Lopes CS, Werneck GL (2005). Estudo Pró-Saúde: características gerais e aspectos metodológicos [The Pro-Saude Study: general characteristics and methodological aspects]. Rev Bras Epidemiol.

[33] Chor D, Faerstein E, Alves MG, de Souza Lopes C (2003). How reproducible is self-reported information on exposure to smoking, drinking, and dietary patterns? Evidence among Brazilian adults in the Pró-Saúde Study. Sao Paulo Med J.

[34] World Health Organization (2008). Waist Circumference and Waist-Hip Ratio.

[35] Alves RFS, Faerstein E (2015). Desigualdade educacional na ocorrência de obesidade abdominal: Estudo Pró-Saúde [Educational inequality in the occurrence of abdominal obesity: Pró-Saúde Study]. Rev Saúde Pública.

[36] Moreno-Betancur M, Latouche A, Menvielle G, Kunst AE, Rey G (2015). Relative index of inequality and slope index of inequality: a structured regression framework for estimation. Epidemiology.

[37] World Health Organization (2013). Handbook on Health Inequality Monitoring: with a special focus on low- and middle-income countries.

[38] Fortin M, Bravo G, Hudon C, Vanasse A, Lapointe L (2005). Prevalence of multimorbidity among adults seen in family practice. Ann Fam Med.

[39] van den Akker M, Buntix F, Metsemakers JF, Roos S, Knottnerus JA (1998). Multimorbidity in general practice: prevalence, incidence, and determinants of co-occurring chronic and recurrent diseases. J Clin Epidemiol.

[40] Britt HC, Harrison CM, Miller GC, Knox SA (2008). Prevalence and patterns of multimorbidity in Australia. Med J Aust.

[41] Galobardes B, Smith GD, Lynch JW (2006). Systematic review of the influence of childhood socioeconomic circumstances on risk for cardiovascular disease in adulthood. Ann Epidemiol.

[42] World Health Organization. Commission on Social Determinants of Health (2008). Health equity through action on the social determinants of health.

[43] Schäfer I, Hansen H, Schön G (2012). The influence of age, gender and socio-economic status on multimorbidity patterns in primary care. First results from the multicare cohort study. BMC Health Serv Res.

[44] Barnett K, Mercer SW, Norbury M (2012). Epidemiology of multimorbidity and implications for health care, research, and medical education: a cross-sectional study. Lancet.

[45] Hudon C, Fortin M, Poitras M-E, Almirall J (2012). The relationship between literacy and multimorbidity in a primary care setting. BMC Fam Pract.

[46] (2013). Reforma da Atenção Primária à Saúde na cidade do Rio de Janeiro - avaliação dos primeiros três anos de Clínicas da Família. Pesquisa avaliativa sobre aspectos de implantação, estrutura, processos e resultados das Clínicas da Família na cidade do Rio de Janeiro.

[47] Starfield B (2002). Atenção primária: equilíbrio entre necessidades de saúde, serviços e tecnologia.

[48] Macinko J, Harris MJ (2015). Brazil’s family health strategy--delivering community-based primary care in a universal health system. N Engl J Med.

[49] (2010). Promoting health and development: closing the implementation gap. Glob Health Promot.

